# Impact of the Configurational Microstructure of Carboxylate-Rich Chitosan Beads on Its Adsorptive Removal of Diclofenac Potassium from Contaminated Water

**DOI:** 10.3390/polym15214274

**Published:** 2023-10-31

**Authors:** Rasha E. Elsayed, Dina Hassanein, Mayyada M. H. El-Sayed, Tarek M. Madkour

**Affiliations:** Department of Chemistry, School of Sciences and Engineering, The American University in Cairo, AUC Avenue, New Cairo 11835, Egypt; r.essam@aucegypt.edu (R.E.E.); dina_h1992@aucegypt.edu (D.H.)

**Keywords:** water treatment, contaminants of emerging concern, grafting, copolymerization, removal

## Abstract

A novel adsorbent–contaminant system was investigated for its ability to remove a contaminant of emerging concern, diclofenac potassium, from contaminated water. Bio-based crosslinked chitosan beads functionalized with poly(itaconic acid) side chains were examined for their potential to remove the emerging contaminant. To evaluate the impact of the polymeric microstructure on its adsorptive capacity, several adsorbent samples were prepared using different combinations of initiator and monomeric concentrations. Fourier Transform Infrared (FTIR) analysis confirmed the crosslinking of the chitosan chains and the incorporation of the carboxylic groups on the surface of the final chitosan beads. After the grafting copolymerization process, an additional peak at 1726 cm^−1^ corresponding to the carboxylic C=O groups of the grafted chains appeared, indicating the successful preparation of poly(IA)-*g*-chitosan. Thermal stability studies showed that the grafting copolymerization improved the thermal stability of the beads. X-ray and Scanning Electron Microscopy confirmed the successful grafting of the itaconic acid on the surface of the beads. The study revealed that the higher the initiator concentration, the greater the number of side chains, whereas the higher the monomeric concentration, the longer the length of these side chains. The adsorption mechanism involved hydrogen bonding to the carboxylic groups of the grafted chains along with n-π* stacking interaction between the amino group of the chitosan and the aromatic rings of diclofenac potassium. The adsorption efficiencies of diclofenac potassium onto the grafted beads were significantly improved compared to the unfunctionalized chitosan beads, reaching values above 90%. The removal efficiency of grafted chitosan increased with an increase in the concentration in the range of 10–30 ppm and then flattened out in the range of 30–50 ppm. The removal efficiencies of 1–50 ppm of DCF ranged between about 75% and 92% for the grafted chitosan and 30–45% for the crosslinked chitosan. Rapid adsorption occurred within 20 min for all grafted sample combinations, and the adsorption kinetics followed a pseudo-second-order model with q_e_ values ranging from 28 to 44.25 g/mg and R^2^ values greater than 0.9915. The results highlight the potential of grafted chitosan beads in removing emerging contaminants from contaminated water without harming the environment.

## 1. Introduction

Despite the global health benefits arising from the research and development of new pharmaceuticals, massive drug manufacturing and overuse have polluted the water environment [1]. Due to the limitations of conventional treatment methods, pharmaceutical residues are becoming one of the major sources of emerging contaminants (EC) in contaminated water [2,3,4]. Despite their low concentrations in contaminated water, pharmaceutical residues can cause adverse impacts on the ecology and human health, such as endocrine disruption, genotoxicity, and aquatic toxicity [5]. Biocomposites obtained from raffia fibers subjected to the hydrothermal carbonization process were used to produce magnetic nanoparticles that can be used as biological adsorbent for the treatment of contaminated water and wastewater and, at the same time, allow for easy separation by applying an external magnetic field [6]. Microorganism-graphene oxide biocomposites were also synthesized and used for Uranium (VI) adsorption from aqueous solutions [7]. However, these systems showed success in the removal of heavy metals but have not been tested yet on emerging contaminants such as diclofenac potassium (DCF).

Diclofenac potassium belongs to the class of non-steroidal anti-inflammatory drugs (NSAID); it is usually used as an antipyretic and it also reduces moderate to severe pain [8]. Recent research verified that DCF causes aquatic ecotoxicity at low levels (>2.00 μg L^−1^) as well as thyroid tumors, hemodynamic changes, and kidney damage in humans upon long-term exposure [9,10,11,12,13]. The European Union’s monitoring list has recently included this drug due to its environmental hazard. Accordingly, novel high-efficiency technologies are being developed to remove DCF contaminants from contaminated water. Among the various treatment processes, adsorption-based technology is characterized by directly removing contaminants without producing toxic intermediates and by its uptake efficacy at low pharmaceutics concentrations [14]. 

Chitosan, a nitrogenous polysaccharide, is a bio-based adsorbent widely used in removing pharmaceutical residues [15,16]. Chitosan materials are typically modified to further enhance their adsorption capacity for pharmaceutical residues [17,18,19,20,21,22,23]. Several research reports discussed DCF removal using chitosan-based materials via adsorption. Grafting copolymerization of chitosan/Fe_3_O_4_ composite with 2-methyl acryloyloxyethyl trimethyl ammonium chloride (DMC), methylmethacrylate (MMA), and acrylic acid (AA) was performed for the removal of DCF from water [24]. All modified composites provided promising results, but DMC/chitosan/Fe_3_O_4_ revealed a maximum capacity of 151.0 mg/g at pH 6. In a recent study, grafting of the surface of cross-linked chitosan beads with polyethyleneimine (PEI) led to the enhancement in the adsorptive removal efficiency toward DCF due to the increase in the number of positive charged amino groups on the adsorbent surface [25]. The maximum adsorption capacity of the PEI-grafted chitosan reached 253.32 mg/g at pH 4.2. A novel core–shell adsorbent composite based on PEI-crosslinked PVA/CS beads reinforced by amino-grafted silica was developed to capture DCF from water [26]. The large number of amino groups (–NH_2_) on the surface of (PVA/CS/SAP@PEI) gel beads improved the adsorption of DCF, attaining a maximum adsorption capacity of 493.81 mg/g at pH 5 via electrostatic attraction forces. In addition, chitosan microspheres were also used in removing DCF by adsorbing the contaminant molecules onto its surface. This is performed via the interaction between its carboxylate groups and the amine groups of the chitosan chains, yielding a maximum adsorption capacity of 5.2 × 10^−4^ mol/g [27]. An interesting study used magnetic ZnFe_2_O_4_/chitosan particles as promising adsorbents for DCF [28]. The developed magnetic particles exhibited high reusability and an adsorption capacity of 188 mg g^−1^. Additional chitosan-based adsorbents were explored, such as activated carbon-chitosan composite, which exhibited an adsorption capacity of 270 mg/g [29], and glutaraldehyde cross-linked magnetic chitosan, which removed 57.5 mg/g of DCF [30]. 

Another member of the carboxylic-rich compounds is Itaconic acid (IA), a dicarboxylate-vinyl organic monomer derived from algae fermentation. It has been used to synthesize resins or modify natural polymers such as chitosan [31,32]. Due to their superior chelating ability with heavy metals in water [33,34,35], these polymers have been employed in contaminated water treatment in powder form [36,37,38,39,40]. 

In this study, carboxylate-rich chitosan is synthesized via surface grafting copolymerization to produce the adsorbent system in a bead form. Unlike other research work, the bead form was chosen in this study for its efficiency in continuous treatment processes and ease of collection after use for regeneration. The epichlorohydrin-crosslinked bio-based chitosan polymer beads are grafted with itaconic acid side chains. A set of samples with varying initiator and monomeric concentrations are synthesized to produce polymers with varying microstructures. The study is further extended to evaluate the impact of the configurational microstructure of these adsorbents on their adsorption capacity for DCF. This novel adsorbent–contaminant system is of major interest since DCF is an emerging contaminant of major concern, and its removal from water bodies without harming the environment is of high priority. The microstructures of the functionalized chitosan samples are evaluated using a variety of spectroscopic techniques. Its thermal behavior is studied using thermogravimetric analysis (TGA), and the adsorption capacity of these polymers is evaluated and explained in terms of their microstructure to establish the relationship between the polymeric structure and its adsorption properties.

## 2. Materials and Methods

### 2.1. Materials

Chitosan (~95% DA) with an average molecular weight (Mn) of 250 kDa was purchased from Pharmchem, Jhajjar, India. Sodium hydroxide (NaOH) and ammonium persulfate (APS) were supplied from Chem Lab, Zedelgem, Belgium. Epichlorohydrin (ECH) and itaconic acid (IA) with a purity of 99% were purchased from Loba Chemie Company, Mumbai, India. Acetic acid was purchased from Sigma Aldrich, Schnelldorf, Germany, and the ethanol absolute anhydrous (≥99.9) was supplied from Carlo Erba, Milano, France. Diclofenac potassium salt (CAS 15307-81-0) was also supplied from Sigma Aldrich (Sigma-Aldrich, Schnelldorf, Germany) with 99.8% purity and 334.24 g mol^−1^ molar mass. 

### 2.2. Methods

#### 2.2.1. Preparation of Chitosan Beads

**Figure 1:**
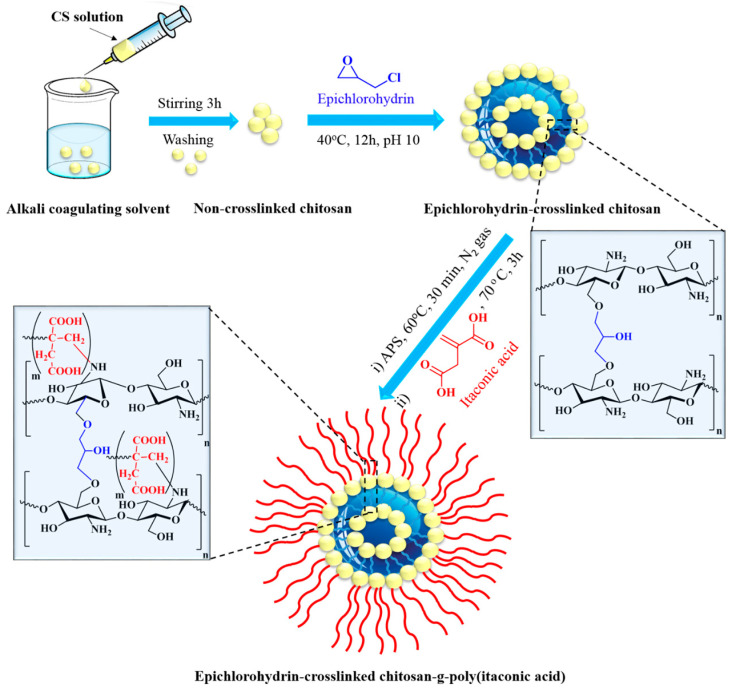
Synthesis procedures of crosslinking of the surface of chitosan beads and grafting copolymerization of the crosslinked beads with itaconic acid. The figure shows a part of the surface of the resultant epichlorohydrin-crosslinked chitosan-*g*-poly(itaconic acid) bead.

Chitosan, CS, (3 g) was dissolved in 100 mL of acetic acid (1% *v*/*v*) in a 250 mL autoclave and stirred constantly until a homogenous solution was obtained. The solution was then added dropwise using a 20-gauge syringe needle into an aqueous 0.5 M NaOH solution. The suspended beads were left in an aqueous NaOH solution for 3 h to neutralize the acid. The beads were filtered and washed thoroughly with distilled water to remove residual NaOH (Figure 1). 

#### 2.2.2. Preparation of Crosslinked Chitosan Beads 

CS beads were crosslinked with ECH by placing the wet beads in 600 mL of ECH solution (2% *v*/*v*). The suspension was heated for 12 h at 50 °C and pH 10. The cross-linked CS beads were filtered and washed several times with distilled water to remove unreacted ECH. The beads were finally dried in a freeze-dryer for two days (Figure 1). 

#### 2.2.3. Preparation of Poly(itaconic Acid)-Grafted Chitosan Beads 

A mass of 1 g of crosslinked CS beads and APS in distilled water (100 mL) was transferred into a 250 mL three-necked flask equipped with a condenser and a magnetic stirrer under a nitrogen blanket. To evaluate the influence of varying the amount of the initiator on the polymeric microstructure, three grafted copolymers were prepared using different amounts of the APS initiator, namely 0.02, 0.06, and 0.1 g. For these three samples, a constant amount of 3 g of itaconic acid monomer was used. In addition, to evaluate the influence of varying the monomer concentration on the polymeric microstructure, three different amounts of itaconic acid monomer, namely 1, 3, and 5 g, were used to produce the other three grafted copolymers. For these samples, the amount of the APS initiator was kept fixed at 0.02 g. The first three samples were given the code names *P*(IA)-*g*-CS-I0.02-M3, *P*(IA)-*g*-CS-I0.06-M3, and *P*(IA)-*g*-CS-I0.1-M3, respectively, which are used throughout the manuscript. The last three samples were given the code names *P*(IA)-*g*-CS-I0.02-M1, *P*(IA)-*g*-CS-I0.02-M3, and *P*(IA)-*g*-CS-I0.02-M5, respectively, which are also used throughout the manuscript (Table 1). In this terminology, “I” refers to the initiator and is followed by the initiator amount in grams, and “M” refers to the monomer and is followed by the monomer amount in grams. Needless to say, the *P*(IA)-*g*-CS-I0.02-M3 sample is common between the two groups. The mixture was stirred for 30 min at 60 °C to facilitate the formation of radicals on CS. The required amount of IA monomer was added gradually to the mixture. The grafting reaction was allowed to take place with continuous stirring and a flow of oxygen-free nitrogen gas for 3 h (Figure 1). The grafted beads were then washed thoroughly with distilled water to remove unreacted reagents and dried in a vacuum oven overnight at 45 °C.

#### 2.2.4. Characterization of the As-Prepared Chitosan Beads

FTIR spectra of the as-prepared CS beads were recorded using a Nicolet FTIR Magna-IR 560 system (Green Bay, WI, USA). Pellets of the crushed beads were mixed with FTIR grade KBr and compressed at a pressure of 1400 kPa to prepare the beads for the analysis experiment. The background spectra were deducted from the sample spectra in the infrared region of 4000–450 cm^−1^ to attribute the net spectra of the samples without interference [41]. 

TGA experiments were conducted using a Thermo Scientific Q Series TGA analyzer to examine the thermal degradation behavior of the crosslinked CS and the influence of grafting copolymerization on its degradation profile. The heating rate of 10 °C min^−1^ was employed under a nitrogen flow rate of 50 mL min^−1^ from 25 °C up to 750 °C. 

The morphological investigation of the cross-linked CS and poly(IA)-g-CS beads was performed using SUPRA 55 LEO SEM instrumentation with high-resolution FEGSEM and a variable pressure option. The elemental composition was quantified using SEM-EDX. 

Zeta potential measurements were performed by dispersing 0.5 mg of the CS beads in 75 mL distilled water and measuring their potential at different pHs using Malvern zeta sizer, Nano Zs, Malvern, UK.

#### 2.2.5. Adsorption of DCF onto the As-Prepared Poly(itaconic Acid)-Grafted Chitosan Beads

A pre-defined concentration of DCF (10, 20, 30, 40, or 50 ppm) was prepared at several pHs and placed in a 15-mL falcon tube along with a pre-determined dose (0.13, 0.266, 0.53, and 1 g/L) of cross-linked CS or grafted CS beads. The mixture was shaken at different intervals in the dark on a Wrist Action shaker (Burrell Scientific, model 75, PA, USA). After shaking, the mixture was centrifuged (HERMLE Laboratory GmbH, Wehingen, Germany) at 12,000 rpm for 10 min, and the absorbance of the supernatant was measured using Jenway 7205 UV-Visible spectrophotometer at a wavelength of 276 nm. The equilibrium concentration was calculated based on a calibration curve, and then the adsorption capacity (q_e_) and removal percentages were calculated using Equations (S1) and (S2), SI. The kinetic uptake experiments were conducted using 50 ppm DCF, 1 g/L adsorbent, at pH 6.5 for 4 h, and the uptake profiles were modeled using the pseudo-first- and pseudo-second-order equations (Equations (S3) and (S4), SI).

#### 2.2.6. Statistical Analysis

The experiments were conducted three times, and the obtained data were presented as the mean ± SD. Statistical analysis was carried out using one-way ANOVA, followed by a Tukey’s multiple comparisons test on all data obtained from the investigation of the removal percentage and q_e_ of the non-grafted and grafted chitosan beads (* *p* < 0.05, ** *p* < 0.01, *** *p* < 0.001, and **** *p* < 0.0001). All tests were calculated using GraphPad Prism Software Version 6.

## 3. Results and Discussion

### 3.1. Fourier Transform Infrared (FTIR) Spectroscopy

**Figure 2:**
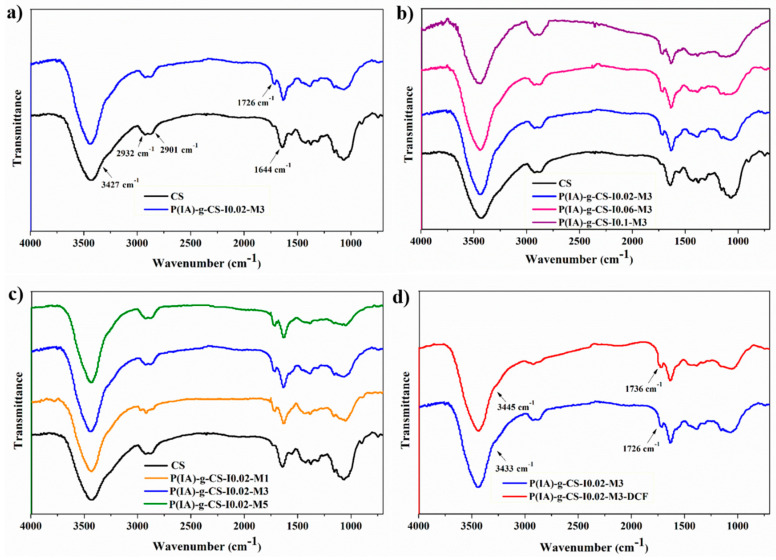
FTIR Spectra of all the prepared samples illustrating the impact of grafting (**a**), initiator concentration (**b**), monomer concentration (**c**), and DCF adsorption (**d**).

The prepared crosslinked CS and poly(IA)-*g*-chitosan beads were characterized via FTIR analysis. In the FTIR spectrum of the crosslinked CS sample (Figure 2a–d), the broadband at 3427 cm^−1^ was assigned to the O-H and N-H functional groups [42]. The appearance of absorption bands at 2932 and 2901 cm^−1^ corresponded to the asymmetric and symmetric stretching vibrations of CH bonds [43]. A sharp peak representing the stretching of the C=O group at 1644 cm^−1^ appeared due to the remaining acetamide group in CS [44,45]. Moreover, the three peaks at 15,551,427, and 1372 cm^−1^ represented NH_2_-bending, C–N bond stretching, and C–H bending, respectively [46]. The bands appearing at 1423 and 1375 cm^−1^ were ascribed to the C–H and secondary –OH bending, respectively [47]. Furthermore, the peaks at 1071 cm^−1^ and 1026 cm^−1^ were attributed to the skeletal vibration of C–O bonds stretching [45]. After the grafting copolymerization process, an additional peak at 1726 cm^−1^ corresponding to the carboxylic C=O groups of the grafted chains appeared, indicating the successful preparation of poly(IA)-*g*-chitosan (Figure 2a) [41,42]. The FTIR spectral analysis was also conducted after DCF adsorption, where 1 g/L of *P*(IA)-*g*-CS-M3 was immersed in a 50 ppm DCF solution at pH 6.5. After adsorption, the distinct peaks of *P*(IA)-*g*-CS-I0.02-M3 at 3433 and 1726 cm^−1^ were slightly blue-shifted to 3445 and 1736 cm^−1^, respectively, indicating the involvement of hydroxyl and carboxylic groups in the adsorption process (Figure 2d) [26]. No significant change was observed in FTIR upon increasing the initiator and monomer concentrations (Figure 2b,c). 

### 3.2. Thermogravimetric Analysis (TGA)

**Figure 3:**
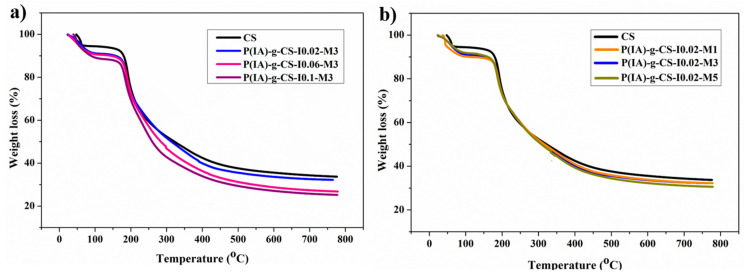
TGA thermograms of all the prepared samples illustrating the impact of initiator concentration (**a**) and monomer concentration (**b**) on the thermal behavior.

The thermal stability of crosslinked CS and poly(IA)-g-CS beads was investigated using TGA techniques. The TGA thermograms (Figure 3a,b) do not show a major change in the degradation profile of the various samples, indicating that any variation in the polymeric configuration and its subsequent impact on the samples’ adsorption capacity is of a physical nature rather than a chemical one. As shown in the figures, the degradation profile of the samples may be divided into two stages of weight loss. The initial weight loss around 104 °C is attributed to the evaporation of water molecules absorbed by the hydrophilic groups of CS [48], with the second stage of weight loss occurring in the range of 170 to 450 °C, which is attributed to both the breakage of the glycosidic bond of the CS backbone and the decoupling of the poly(itaconic acid) side chains from the CS backbone. Since the decoupling of the poly(itaconic acid) side chains occurs at lower temperatures than that of CS pyrolysis, a slight shift in the thermal degradation of the grafted CS beads is observed [49,50,51]. Interestingly, the degradation temperature at which 50% weight loss occurs is observed to increase with an increase in the initiator concentration (Figure 3a). This is because the increase in the initiator concentration increases the number of side chains, which would require higher energies to decouple the side chains from the main backbone of the polymeric network. No significant effect on thermal degradation is shown upon increasing the monomer concentration (Figure 3b), indicating that the increase in the length of the side chains resulting from the increase in the monomer concentration bears no effect on the degradation profile. Weight loss after 450 °C is related to the carbonization of the polymeric network [47,50].

### 3.3. Morphological Screening 

**Figure 4:**
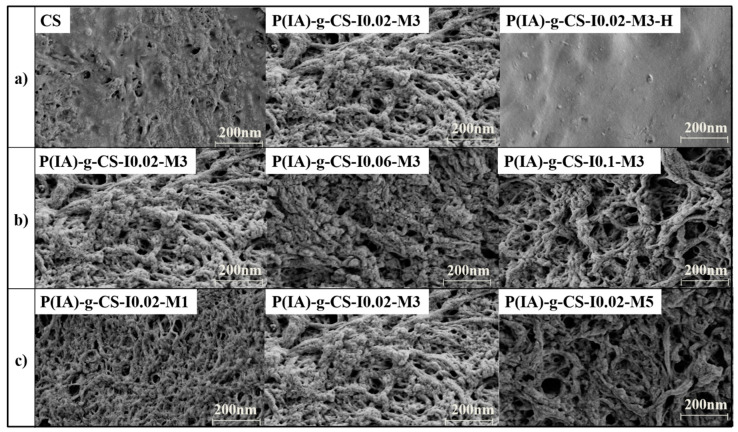
SEM images of all the prepared samples illustrating the impact of grafting and drying (**a**), initiator concentration (**b**), and monomer concentration (**c**) on the bead surface.

To study the influence of surface grafting copolymerization of CS on the surface texture and morphology of the various samples, their SEM images were examined. Figure 4a–c shows the SEM images of the crosslinked CS and *P*(IA)-*g*-CS samples. 

Unlike the non-grafted crosslinked chitosan beads, the surface of the grafted CS appeared to be more porous, rougher and showed a spherulite-like structure, as shown in Figure 4a. This may be attributed to the existence of a large number of side chains on the surface of the grafted CS beads, which would form hydrogen bonding with absorbed water molecules. Upon freeze-drying, water evaporation leaves tiny cavities. To confirm this hypothesis, *P*(IA)-*g*-CS-I0.02-M3 sample was dried under vacuum, and its SEM images were compared to those of the samples dried using a freeze-dryer. The sample dried under vacuum was given the code name *P*(IA)-*g*-CS-I0.02-M3-H, as shown in Figure 4a. Interestingly, it can be observed that the surface of such a sample is smooth and homogenous, indicating the ability of the freeze-dried grafted samples to absorb a large amount of water due to its rough surface. Varying the initiator and the monomer concentrations has no appreciable effect on the morphology of the grafted samples, as shown in Figure 4b,c, respectively.

### 3.4. Elemental Composition 

EDX analysis confirmed the presence of three intense peaks of C, N, and O, which signified the main elements in the crosslinked CS and poly(IA)-*g*-CS samples (Table 2). As shown in Table 2, the total content of C and O (CK+OK) of CS increased after its modification. The increase in the total contents of C and O confirms the successful grafting of IA on the surface of ECH-crosslinked CS beads. Interestingly, the increase in the total contents of C and O is observed upon increasing the monomer concentration (Table 2). On the other hand, no appreciable change in the total content of C and O is observed with the increase in the initiator concentration (Table 2). 

### 3.5. X-ray Photoelectron Spectroscopy (XPS) Analysis

**Figure 5:**
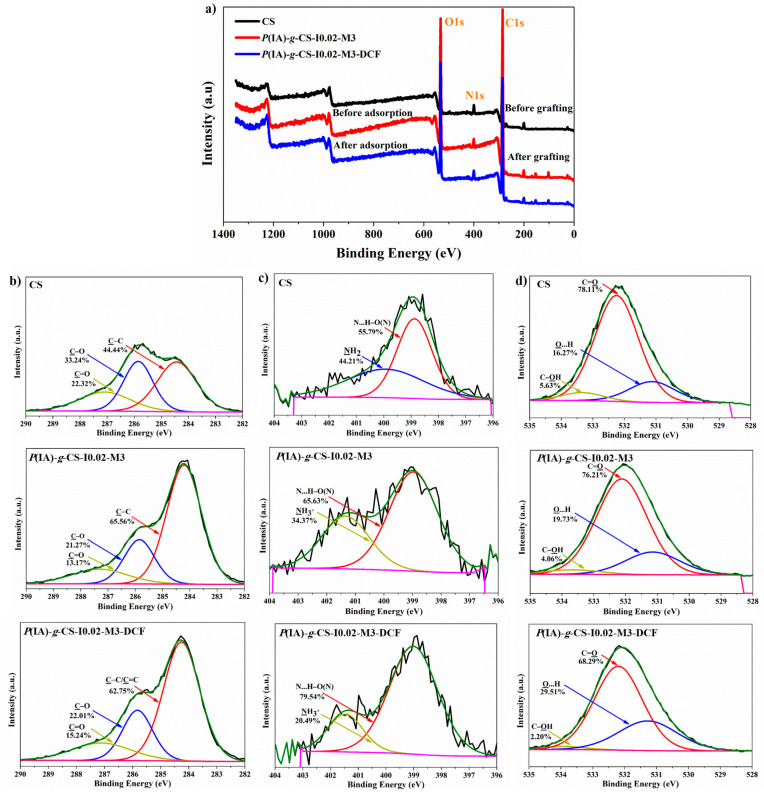
XPS spectra of all the prepared samples. (**a**) Wide scan, (**b**) C1s spectra, (**c**) N1s spectra, and (**d**) O1s spectra.

Sample *P*(IA)-*g*-CS-I0.02-M3 was the chosen grafted sample in the XPS study because it is common between the varying initiator concentration and varying monomer concentration groups. The survey XPS spectra of CS, *P*(IA)-*g*-CS-I0.02-M3, and *P*(IA)-*g*-CS-I0.02-M3-DCF revealed the three elements contained in the tested adsorbents, namely O, N, and C (Figure 5a). As shown in Figure 5a, three peaks are observed in the C1s spectrum of CS at 284.43, 285.85, and 287.09 eV corresponding to C–O, C–N, and C=O groups, respectively [52,53]. Interestingly, the proportion of C–C distinctly increased from 44.44% to 65.56% after grafting (Figure 5b) due to the increase in the content of C–C on the surface of grafted CS beads. In contrast, the proportions of C–O and C=O significantly declined from 33.24% and 21.27% to 22.32% and 13.17%, respectively. This indicates the formation of hydrogen bonding between the carboxylic group of grafted chains and the chitosan backbone after grafting, as shown in the C1s spectra of *P*(IA)-*g*-CS-I0.02-M3 (Figure 5b). After DCF adsorption (Figure 5b), a minor decrease in the C–C peak was observed. This might be attributed to the binding of C–C and C=C of DCF aromatic rings with the nitrogen atom of the amino group on the CS chain through n–π* stacking. XPS technique is also useful in determining the valence states of nitrogen in the samples as depicted in the high-resolution XPS spectra of N1s regions for CS, *P*(IA)-*g*-CS-I0.02-M3 and *P*(IA)-*g*-CS-I0.02-M3-DCF samples shown in Figure 5c. Two peaks related to different nitrogen-containing groups are deconvoluted in the N1s spectra [54]. In the case of CS, the peaks at binding energies of 399.92 and 398.87 eV originate from the hydrogen-bonded nitrogen atoms [N…H–O(N)] and a primary amino group (NH_2_), respectively. A new –NH_3_^+^ peak appears in the N1s spectrum of *P*(IA)-*g*-CS-I0.02-M3 at 401.34 eV after grafting, signifying the change in the state of primary amino groups during the grafting process. The protonated amino groups (NH_3_^+^) are critical for the electrostatic attraction with the carboxylic groups of the grafted chains. The intensity of the N…H–O(N) peak also increased from 55.79% to 65.63%, probably due to the formation of hydrogen bonding between carboxylic groups of the grafted chains and the amino group of chitosan [55]. Interestingly, after DCF adsorption, the N1s spectrum of *P*(IA)-*g*-CS-I0.02-M3 demonstrates a clear increase in the intensity of N…H–O(N) as well as a significant decline in the intensity of NH_3_^+^ peak, suggesting that hydrogen bonding possibly took place between the carboxylic group of DCF and the amino group of chitosan [54,55]. As shown in Figure 5d, the O1s high-resolution spectrum of CS exhibits three peaks at 531.12, 532.25eV, and 533.33eV, corresponding to hydrogen-bonded oxygen atoms (O…H), oxygen atoms of the carboxylic group (C=O), oxygen atoms of hydroxyl group binding to either the carbon atoms of the chitosan backbone or the carbon atoms of the crosslinker side chains (C–OH) [55,56,57]. The intensities of O…H barely increased from 16.27% to 19.73% after grafting, as presented in *P*(IA)-*g*-CS-I0.02-M3 spectrum. In contrast, the proportions of C=O and C–OH decreased slightly from 78.11% and 5.63% to 76.21% and 4.06%, respectively. This is evidence of the presence of hydrogen bonding between carboxylic groups of the grafted side chains and hydroxyl groups binding to either the carbon atoms of the chitosan backbone or the carbon atoms of the crosslinker side chains as well as between carboxylic groups with each other [55]. In addition, the C=O of the remaining acetyl groups contributed little to the increase in hydrogen bonding [58]. Similar trends are observed after DCF adsorption (*P*(IA)-*g*-CS-I0.02-M3-DCF). After the adsorption of DCF, a significant increase in the proportion of O…H peak from 19.73% to 29.51% as well as a noticeable decline in the proportions of C=O and C–OH to 68.29% and 2.20% are observed. These results confirm the participation of the C–OH and C=O groups in forming O⋯H hydrogen bonds during the adsorption of DCF on the surface of the grafted CS beads, as reported previously [55].

### 3.6. Adsorption of DCF under Different Operational Parameters

**Figure 6:**
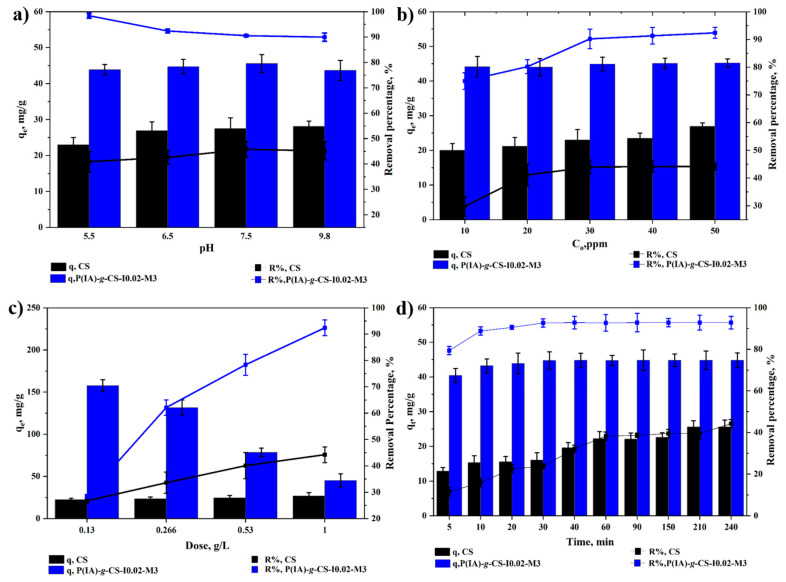
Impact of grafting of chitosan on equilibrium uptake capacity and removal percentage of DCF at different pHs (**a**), initial DCF concentrations (**b**), adsorbent dose (**c**), and time (**d**). Experimental conditions are 1 g/L adsorbent dose and 50 ppm in (**a**); 1 g/L and pH 6.5 in (**b**); 50 ppm and pH 6.5 in (**c**); and 1 g/L, 50 ppm, and 25 °C in (**d**).

**Figure 7:**
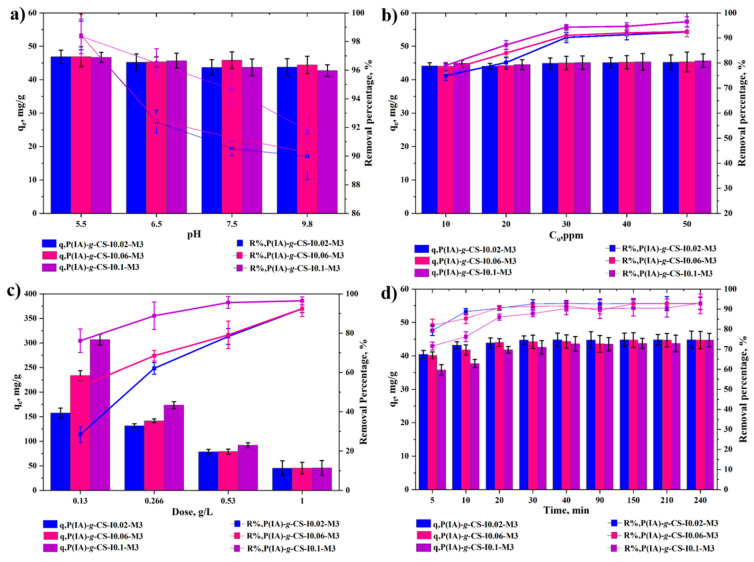
Impact of initiator concentration on equilibrium uptake capacity and removal percentage of DCF at different pHs (**a**), initial DCF concentrations (**b**), adsorbent dose (**c**), and time (**d**). Experimental conditions are 1 g/L adsorbent dose and 50 ppm for (**a**); 1 g/L, pH 6.5 for (**b**); 50 ppm and pH 6.5 for (**c**); and 1 g/L, 50 ppm, and 25 °C for (**d**).

**Figure 8:**
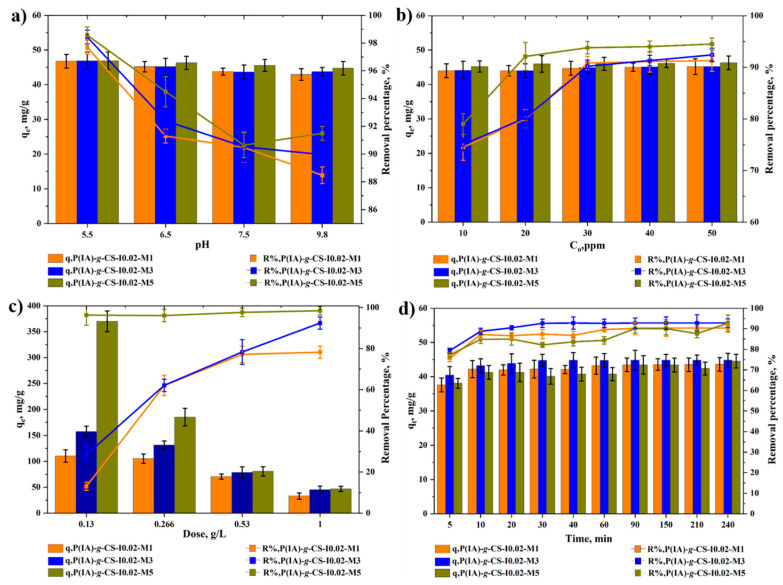
Impact of monomer concentration on equilibrium uptake capacity and removal percentage of DCF at different pHs (**a**), initial DCF concentrations (**b**), adsorbent dose (**c**), and time (**d**). Experimental conditions are 1 g/L adsorbent dose and 50 ppm for (**a**); 1 g/L, pH 6.5 for (**b**); 50 ppm and pH 6.5 for (**c**); and 1 g/L, 50 ppm, and 25 °C for (**d**).

The influence of both the grafting and the variation in the initiator and monomer concentrations on the polymeric structure–property relationship is studied with regard to the equilibrium adsorption capacities and DCF removal efficacies of the different samples. This is conducted to explore the best conditions for the maximum uptake of the DCF from contaminated water in a rapid and effective manner (Figure 6, Figure 7 and Figure 8). The equilibrium adsorption capacities (q_e_) and removal efficiencies (R%) of DCF onto crosslinked CS and *P*(IA)-*g*-CS-I0.02-M3 grafted chitosan beads are shown in Figure 6 as a function of pH (Figure 6a), initial DCF concentration (Figure 6b), adsorbent dose (Figure 6c), and time (Figure 6d). As shown in Figure 6a, both q_e_ and R% slightly increase with increasing pH. This is because most of the primary amine groups of CS below pH of 6.5 are protonated (pK_a_~6.5) [24,36]. These pK_a_ values indicate that the amine groups are positively charged and, hence, interact electrostatically with the negatively charged DCF (pK_a_~4.2) [59]. However, it is anticipated that this interaction is weak since CS is slightly positive, as shown in the zeta potential diagram, Figure S1, possibly due to the formation of a hydrogen bonding network between the protonated amine groups and the water molecules. Above pH 6.5, the amine groups are mostly non-protonated, and CS acquires a neutral charge (Figure S1); thus, the interaction is likely to take place via other mechanisms like hydrogen bonding or dispersion forces. As for the grafted CS beads, no appreciable change in either q_e_ or R% is observed with varying pH values. It can be envisaged that the electrostatic repulsion forces between the negatively charged grafted CS beads, shown in Figure S1, and the negatively charged DCF have counteracted the effect of other interaction mechanisms. As shown in Figure 6b, both q_e_ and R% of the crosslinked CS increase with an increase in the initial concentration of DCF as a result of the increase in the mass transfer rate arising from the concentration gradient [49,52,53] till the point of saturation of the active sites where a plateau is observed [26,52]; however, q_e_ of the grafted CS does not appreciably change with concentration. The adsorption efficiencies of diclofenac potassium onto the grafted beads were significantly improved compared to the unfunctionalized chitosan beads, reaching values above 90% with rapid adsorption occurring within 20 min for all grafted sample combinations. The removal efficiency of grafted CS increases with increasing concentration in the range of 10–30 ppm, and then flattens out in the range of 30–50 ppm. The removal efficiencies of 1–50 ppm of DCF ranged between about 75% and 92% for the grafted CS and between 30 and 45% for the crosslinked CS. By increasing the adsorbent dose, q_e_ and removal percent increase for the crosslinked CS due to an increase in the adsorption sites (Figure 6c). However, q_e_ decreases for the grafted CS, indicating an agglomeration of the particles, which reduces the surface area available for adsorption [26]. The removal, however, increases with an increase in the adsorbent dose [53]. The time profiles for the adsorption of DCF onto the crosslinked CS and grafted CS (Figure 6d) manifest faster adsorption kinetics onto the grafted beads. Adsorption onto the grafted beads reaches equilibrium in less than 30 min, which is almost half the time taken for the adsorption onto the crosslinked CS. The rougher surface of the grafted beads may have increased the surface area available for adsorption and consequently enhanced the rate. Generally, adsorption onto the grafted beads under different investigated parameters yields higher q_e_ and removal efficiency than adsorption onto crosslinked CS due to the grafted carboxylate-rich side chains, which can be attributed to the higher functionality of the grafted beads by virtue of the functional groups grafted on their surface and the rougher surface of the grafted beads, illustrated in the SEM images shown earlier, and thus resulting in improved adsorption efficacies.

Figure 7 shows the effect of initiator concentration on the performance of the grafted CS adsorbents under different conditions of pH (Figure 7a), initial DCF concentration (Figure 7b), adsorbent dose (Figure 7c), and time (Figure 7d). In general, the same adsorption trends exhibited by *P*(IA)-*g*-CS-I0.02-M3 in Figure 6 are shown by *P*(IA)-*g*-CS-I0.06-M3 and *P*(IA)-*g*-CS-I0.1-M3, while the percent removal increased with an increase in the initiator concentration. The increase in the latter apparently increased the number of initial copolymerization sites on the main CS backbone and, therefore, increased the number of side chains [60], which in turn caused an increase in the adsorbent functional groups and improved their capacity for adsorption and its ability to remove greater amounts of the contaminant molecules. The q_e_, however, was not appreciably affected by the increase in the pH for the different samples prepared using different initiator concentrations (Figure 7a). The same behavior can be observed for q_e_ at different DCF concentrations (Figure 7b). However, at low adsorbent doses, the increase in the initiator concentration caused a noticeable increase in the q_e_ values. At higher adsorbent doses, the effect is minimal since, at this point, higher amounts of the adsorbent beads exhibit the agglomeration effect, and, therefore, increasing the number of side chains is offset by the shear presence of a large number of the side chains. Furthermore, the kinetic profiles in Figure 7d show that the adsorption onto the grafted adsorbents with the highest initiator concentration undergoes the slowest kinetics as a result of the aforementioned agglomeration effect. With regard to the removal efficiencies of the adsorbents grafted at different initiator concentrations, they ranged between about 75 and 96% at the DCF concentration range of 10–50 ppm (Figure 7b) and 1 g/L adsorbent dose.

The effect of monomer concentration is illustrated in Figure 8. Again, the same adsorption trends exhibited in Figure 7 and Figure 8 are shown for the effects of pH (Figure 8a), initial DCF concentration (Figure 8b), adsorbent dose (Figure 8c), and time (Figure 8d). Generally, the removal increases with increasing monomer concentration, whereas q_e_ is not appreciably affected except at low adsorbent doses; it increases with an increase in the monomer concentration. Increasing the latter naturally increases the length of the carboxylate-rich side chains and, thus, its capacity to remove more contaminant molecules [61]. In terms of q_e_, the longer the side chains, the greater the chance for the chains present on the surface of the adsorbent beads to entangle and agglomerate, resulting in a modest effect on the equilibrium adsorption capacities. This impact of the polymeric configurational microstructure on the adsorption capacities of the adsorbent beads is also shown in Figure 8d, and the beads grafted with the highest monomer concentrations show the slowest adsorption kinetics. Regarding the removal efficiencies of the adsorbents grafted at different monomer concentrations, they ranged between about 75 and 95% at the DCF concentration range of 10–50 ppm (Figure 8b) and 1 g/L adsorbent dose.

### 3.7. Statistical Analysis

**Figure 9:**
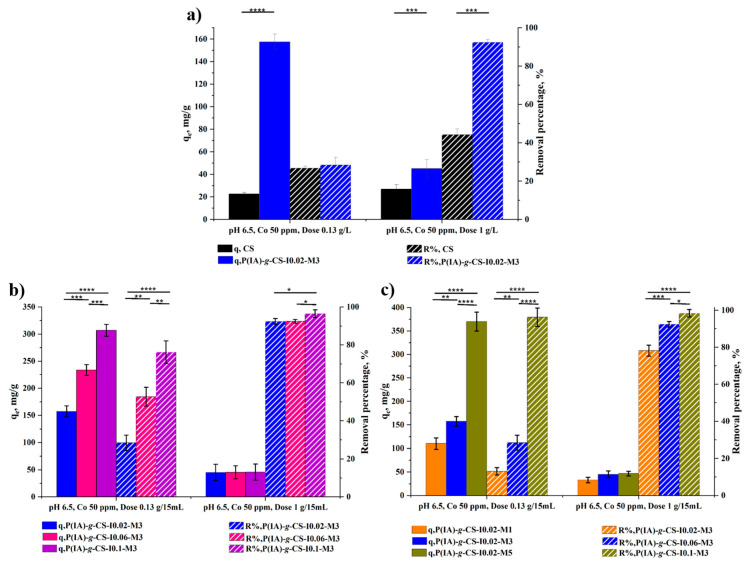
Impact of grafting (**a**), initiator concentration (**b**), and monomer concentration (**c**) on the removal of DCF and q_e_ under the selected conditions: pH 6.5, C_o_ 50 ppm, and dose 0.13 g/L; and pH 6.5, C_o_ 50 ppm, and dose 1 g/L. Data represent the mean ± SD. *p* values are for one-way ANOVA, followed by Tukey’s multiple comparison test. (* *p* < 0.05, ** *p* < 0.01, *** *p* < 0.001, and **** *p* < 0.0001).

The data statistical analysis was conducted under two selected conditions: pH 6.5, C_o_ 50 ppm, and dose 0.13 g/L; and pH 6.5, C_o_ 50 ppm, and dose 1 g/L. This was to explore the influence of the grafting copolymerization, and the variation in the initiator and monomer concentrations on the equilibrium adsorption capacities (q_e_) and removal efficiencies (R%). The resultant statistical data in Figure 9a indicate that the grafting copolymerization significantly increased q_e_ under the two selected conditions (up to **** *p* < 0.0001). No significant difference between the non-grafted and grafted adsorbents in removing DCF under the low adsorbent dose conditions was detected. However, the influence of grafting copolymerization was clearly observed in the removal of DCF at the high adsorbent dose (*** *p* < 0.001). On the other hand, the impact of initiator concentration on the removal and q_e_ under the selected condition with the lowest adsorbent dose (pH 6.5, C_o_ 50 ppm, and dose 0.13 g/15mL) is represented in Figure 9b. In general, a significant effect on both the percent removal and q_e_ is observed upon increasing the initiator concentration (up to **** *p* < 0.0001). Nevertheless, q_e_ is not significantly affected by the initiator concentration at the high adsorbent dose. As for the removal, no significant change was observed upon increasing the initiator concentration from 0.02 g to 0.06 g (* *p* > 0.05), whereas a significant change occurred when the initiator concentration increased to 0.1 g (* *p* < 0.05). As depicted in Figure 9c, the monomer concentration had a significant effect on removing DCF at both experimental conditions (up to **** *p* < 0.0001). The same applied to q_e_ at the lowest adsorbent dose. However, at the highest adsorbent dose, no significant change in q_e_ was noticed (* *p* > 0.05) due to particle agglomeration resulting from the apparent increase in the length of the side chains with an increase in the monomer concentration, which caused these polymeric chains to entangle and lose part of its adsorption capacity. The chain entanglement, thus, acts as a barrier that prevents the contaminant molecules from reaching and attaching to the carboxylate functional groups. 

### 3.8. Modeling of the DCF Adsorption on the Polymeric Adsorbents

#### 3.8.1. Kinetic Modeling

The time profiles in Figure 6, Figure 7 and Figure 8d were fitted to the pseudo-first and pseudo-second order model equations (Equations (S3) and (S4), respectively), and the kinetic linear plots are shown in Figures S2 and S3, respectively. Clearly, better fits are obtained using the pseudo-second-order model, as evident from its higher correlation factors (R^2^) relative to the pseudo-first-order model. The kinetic parameters obtained from the pseudo-second-order fits (Figure S3) are compiled in Table 3. Comparing the various second-order rate constants (k_2_) of the adsorbents, it can be deduced that the rate of DCF adsorption onto non-grafted CS is the lowest among all other adsorbents. This can be attributed to the rough surface of the grafted beads resulting from the side chains of different numbers and lengths. It is interesting to observe that the sample with the highest monomer concentration shows a slightly lower equilibrium adsorption capacity, Table 3. Although the chain length increases with increasing monomer concentration, which should result in enhancing the rate of adsorption, very long chains can become entangled due to their flexibility. This can eventually lead to slowing down the rate of adsorption on the chain surface as some functional groups become inaccessible to DCF.

#### 3.8.2. Isotherm Modeling

Adsorption isotherms for all the investigated adsorbate–adsorbent systems were fitted to Langmuir (Equation (S5)) and Freundlich (Equation (S6)) models and the linear plots are shown in Figures S4–S6. The isotherm parameters are compiled in Table 4, along with the correlation factors (R^2^). Clearly, the crosslinked non-grafted chitosan shows the least maximum adsorption capacity (q_m_) among the tested systems, being 25% less than the capacities of the grafted polymers. No appreciable effect is, however, shown for the initiator or monomer concentration on the capacity. These findings are consistent with the results obtained earlier in the kinetic and equilibrium studies under different operating parameters.

### 3.9. Mechanisms of the Graft Copolymerization and the Adsorption Processes

**Figure 10:**
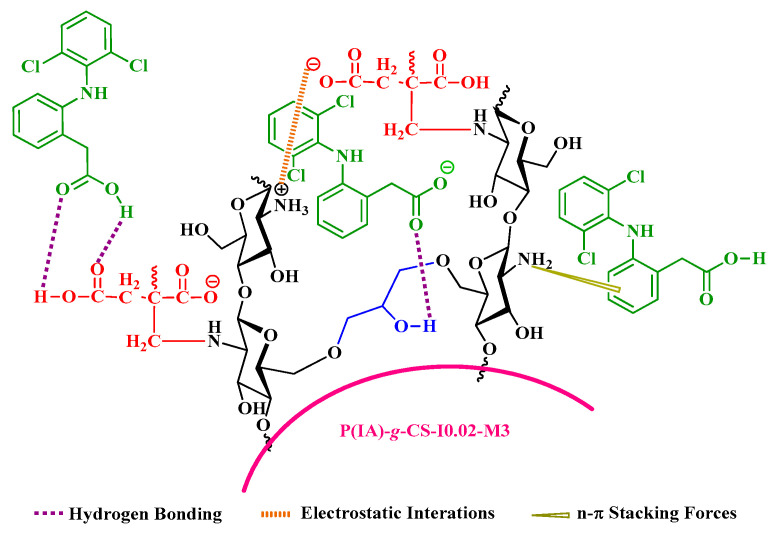
Schematic diagram of some possible mechanisms for polymer grafting and DCF adsorption at different pHs. DCF is negatively charged at pH > 4.2, and neutral at pH < 4.2.

Based on the results of FTIR and XPS, carbonyl and hydroxyl groups from either the side of DCF or the side of the grafted CS beads play a role in adsorption via hydrogen bonding (Figure 10). In addition, n-π* stacking interaction could take place between the nitrogen atom of the amino group on the CS chain and π-bonds in the aromatic rings of DCF [62]. The plausible mechanisms of DCF adsorption on the PIA-*g*-CS beads are schematically illustrated in Figure 10.

### 3.10. Regeneration

**Figure 11:**
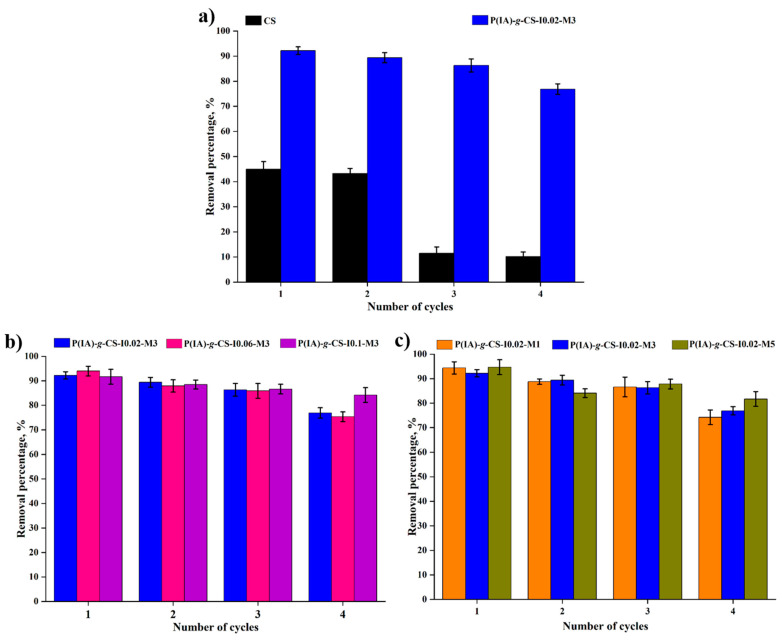
Impact of grafting (**a**), initiator concentration (**b**), and monomer concentration (**c**) on the four cycles of the adsorption–desorption of DCF on the surface of poly(itaconic acid)-grafted chitosan beads.

Regeneration of the adsorbents (Figure 11) shows that the grafted beads can be successfully reused for four consecutive cycles, with their average removal dropping by less than 15% in the fourth cycle. The removal efficacy of crosslinked CS, however, declines by more than 80% in the third cycle, hence indicating the positive influence grafting copolymerization has on the regeneration of the biodegradable adsorbents.

## 4. Conclusions

A series of non-grafted and poly(itaconic acid)-grafted crosslinked chitosan materials were synthesized and examined for their potential to remove contaminants of emerging concern, such as DCF. The investigation also examined the thermal stability, surface morphology, and adsorption properties of prepared adsorbents. Fourier Transform Infrared (FTIR) and X-ray photoelectron spectroscopy (XPS) analyses identified the distinct functional groups and thus confirmed the chemical structures of the polymers. After the grafting copolymerization process, an additional peak at 1726 cm^−1^ corresponding to the carboxylic C=O groups of the grafted chains appeared, indicating the successful preparation of poly(IA)-*g*-chitosan. The appearance of absorption bands and shifts in peak positions provided evidence for successful grafting copolymerization. Thermal analysis using Thermogravimetric Analysis (TGA) revealed that changes in the degradation profile were more likely attributed to physical rather than chemical variations in the polymer configuration. The presence of side chains altered the degradation behavior, leading to a shift in the thermal degradation temperature. Surface morphology studies utilizing Scanning Electron Microscopy (SEM) displayed significant differences between the non-grafted and grafted chitosan beads. The grafted beads exhibited a more porous and rougher surface, attributed to the presence of side chains that formed hydrogen bonds with absorbed water molecules. Energy Dispersive X-ray Spectroscopy (EDX) analysis supported successful grafting, with increased carbon and oxygen contents on the grafted chitosan beads’ surfaces. XPS investigations revealed changes in the chemical composition of the grafted chitosan beads. Notably, the increased presence of C-C bonds and hydrogen bonding interactions were observed due to the grafting of poly(itaconic acid) side chains onto the chitosan backbone. These interactions played a pivotal role in the adsorption of DCF onto the grafted chitosan beads. Equilibrium adsorption studies highlighted the enhanced adsorption capacities and removal efficiencies of DCF by the grafted chitosan beads compared to the non-grafted ones. The adsorption process was influenced by pH, initial DCF concentration, adsorbent dose, and time. The presence of carboxylate-rich side chains and the rough surface of the grafted beads contributed to an improved adsorption efficacy. The impact of grafting copolymerization, initiator concentration, and monomer concentration on the adsorption capacities and removal efficiencies was also investigated and indicated their critical role in the adsorption performance. The adsorption efficiencies of diclofenac potassium onto the grafted beads were significantly improved compared to the unfunctionalized chitosan beads, reaching values above 90% with rapid adsorption occurring within 20 min for all grafted sample combinations. The removal efficiency of grafted CS increased with an increase in the concentration in the range of 10–30 ppm and then flattened out in the range of 30–50 ppm. The removal efficiencies of 1–50 ppm of DCF ranged between about 75% and 92% for the grafted CS and between 30 and 45% for the crosslinked CS. The kinetic analysis supported the pseudo-second-order model for the adsorption process with q_e_ values ranging from 28 to 44.25 g/mg and R^2^ values greater than 0.9915, highlighting an enhanced adsorption rate of the grafted beads. The carbonyl and hydroxyl groups facilitated the hydrogen bonding interactions between the adsorbent and DCF. Additionally, n–π* stacking interactions between the chitosan and the DCF molecules played a significant role in the adsorption mechanism. The regeneration study demonstrated the potential for reusing the grafted beads for multiple cycles, with only minor reductions in adsorption efficiency. This confirmed the practicality and durability of the grafted chitosan beads as effective adsorbents for removing DCF from contaminated water. Understanding the impact of the configurational microstructure of the carboxylate-rich chitosan on its adsorptive removal has led to the development of various mechanisms these samples use to remove contaminants of emerging concern from contaminated water. The results hold significance for developing efficient and sustainable solutions for water treatment and contaminant removal, with potential applications in environmental remediation and water purification processes. Future work might extend to employing these new adsorbent systems for removing other contaminants from contaminated water and thus providing a low-cost and facile method for treating contaminated water, especially in remote low-income areas.

## Figures and Tables

**Table 1:** Mass composition of non-grafted and poly(itaconic acid)-grafted chitosan beads.

Samples	Chitosan	APS Initiator	IA Monomer
CS	1	0	0
*P*(IA)-*g*-CS-I0.02-M3 *	1	0.02	3
*P*(IA)-*g*-CS-I0.06-M3	1	0.06	3
*P*(IA)-*g*-CS-I0.1-M3	1	0.1	3
*P*(IA)-*g*-CS-I0.02-M1	1	0.02	1
*P*(IA)-*g*-CS-I0.02-M5	1	0.02	5

* The *P*(IA)-*g*-CS-I0.1-M3 sample is common between the varying initiator concentration and monomer concentration groups.

**Table 2:** EDX for CS, *P*(IA)-*g*-CS-I0.02-M3, *P*(IA)-*g*-CS-I0.06-M3, *P*(IA)-*g*-CS-I0.1-M3, *P*(IA)-*g*-CS-I0.02-M1, and *P*(IA)-*g*-CS-I0.02-M5 samples.

Samples	Content (wt%)			
	CK	NK	OK	CK + OK
CS	26.66	17.85	55.49	82.15
*P*(IA)-*g*-CS-I0.02-M3	28.02	13.22	58.76	86.78
*P*(IA)-*g*-CS-I0.06-M3	24.75	12.48	61.07	86.52
*P*(IA)-*g*-CS-I0.1-M3	26.45	13.70	61.55	86.30
*P*(IA)-*g*-CS-I0.02-M1	28.03	13.90	58.07	86.10
*P*(IA)-*g*-CS-I0.02-M5	28.34	11.91	59.75	88.09

**Table 3:** Kinetic parameters for the adsorption of DCF on the as-synthesized adsorbents.

Adsorbents	q_e_, mg/g	k_2_, g·mg^−1^·min^−1^	R^2^
CS	28.01	0.002	0.9915
*P*(IA)-CS-*g*-I0.02-M3	44.84	0.065	1.0000
*P*(IA)-CS-*g*-I0.06-M3	44.84	0.031	0.9999
*P*(IA)-CS-*g*-I0.1-M3	44.44	0.015	0.9998
*P*(IA)-CS-*g*-I0.02-M1	43.67	0.026	1.0000
*P*(IA)-CS-*g*-I0.02-M3	44.84	0.065	1.0000
*P*(IA)-CS-*g*-I0.02-M5	44.25	0.008	0.9992

**Table 4:** Isotherm parameters for the tested adsorbents.

Adsorbent	Langmuir Isotherm			Freundlich Isotherm		
	q_m_ (mg/g)	k_d_ (L/mg)	R^2^	K_f_ (mg/g)(L/mg)^n^	1/n	R^2^
CS	35.97	3.42	0.972	12.49	0.334	0.951
*P*(IA)-*g*-CS-I0.02-M3	48.31	0.19	0.999	41.69	0.078	0.967
*P*(IA)-*g*-CS-I0.06-M3	48.08	0.14	0.997	42.27	0.068	0.951
*P*(IA)-*g*-CS-I0.1-M3	47.85	0.12	0.999	43.35	0.050	0.957
*P*(IA)-*g*-CS-I0.02-M1	47.39	0.13	0.999	42.56	0.058	0.952
*P*(IA)-*g*-CS-I0.02-M5	48.08	0.14	0.999	43.35	0.049	0.958

## Data Availability

Available within the manuscript and supplementary file.

## Supplementary Materials

The following supporting information can be downloaded at: https://www.mdpi.com/article/10.3390/polym15214274/s1. Refs. [2,63,64,65,66] are cited in the supplementary materials.
