# Supplementary material for: Impact of the Configurational Microstructure of Carboxylate-Rich Chitosan Beads on Its Adsorptive Removal of Diclofenac Potassium from Contaminated Water
Source: Polymers (Basel). 2023 Oct 31;15(21):4274. doi: 10.3390/polym15214274 (PMC10649765; doi:10.3390/polym15214274)
Supplement: Supplementary file 1 [file polymers-15-04274-s001.zip › polymers-2654890-supplementary.pdf]

Impact of the configurational microstructure of carboxylate-rich chitosan beads on its adsorptive removal of diclofenac Potassium from contaminated water

Rasha E. Elsayed, Dina Hassanein, Mayyada M. H. El-Sayed * and Tarek M. Madkour *
Department of Chemistry, School of Sciences and Engineering, The American University in Cairo, AUC Avenue, New Cairo 11835, Egypt; r.essam@aucegypt.edu (R.E.E.); dina_h1992@aucegypt.edu (D.H.)
* Correspondence: mayyada@aucegypt.edu (M.M.H.E.-S.); tarekmadkour@aucegypt.edu (T.M.M.)

Adsorption calculations[1, 2]

$$\%\,Removal = \frac{(C_0 - C_e)}{C_0} \times 100 \qquad [S1]$$

$$q_e = \frac{(C_0 - C_e)\,V}{m} \qquad [S2]$$

where m and V are mass of polymer in g and volume of DCF solution in L, respectively, while C_o is the initial concentration of DCF, and C_e is the equilibrium concentration of DCF in mg/L.

Kinetic modeling [3, 4, 5]

$$\log(q_e - q_t) = \log(q_e) - \frac{k_1 t}{2.303} \qquad [S3]$$

$$\frac{t}{q_t} = \frac{1}{k_2 q_e^2} + \frac{1}{q_e}\,t \qquad [S4]$$

where k_1 and k_2 are the rate constants for the pseudo-first order and pseudo-second order kinetic models, respectively and q_t is the adsorption capacity at time, t.

Equilibrium modeling[1,2, 3]

Langmuir isotherm $$\frac{C_e}{q_e} = \frac{C_e}{q_m} + \frac{K_d}{q_m} \qquad [S5]$$

Freundlich isotherm $$\log q_e = log\,K_f + \frac{1}{n} log C_e \qquad [S6]$$

where q_e and q_m are the equilibrium and maximum adsorption capacities, C_e is the equilibrium concentration, and K_d is the dissociation constant.

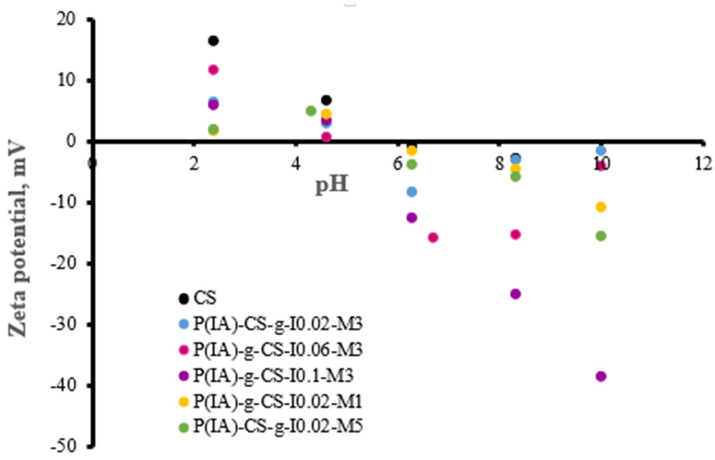

Figure S1 Zeta potential for the as-synthesized adsorbents as a function of pH.

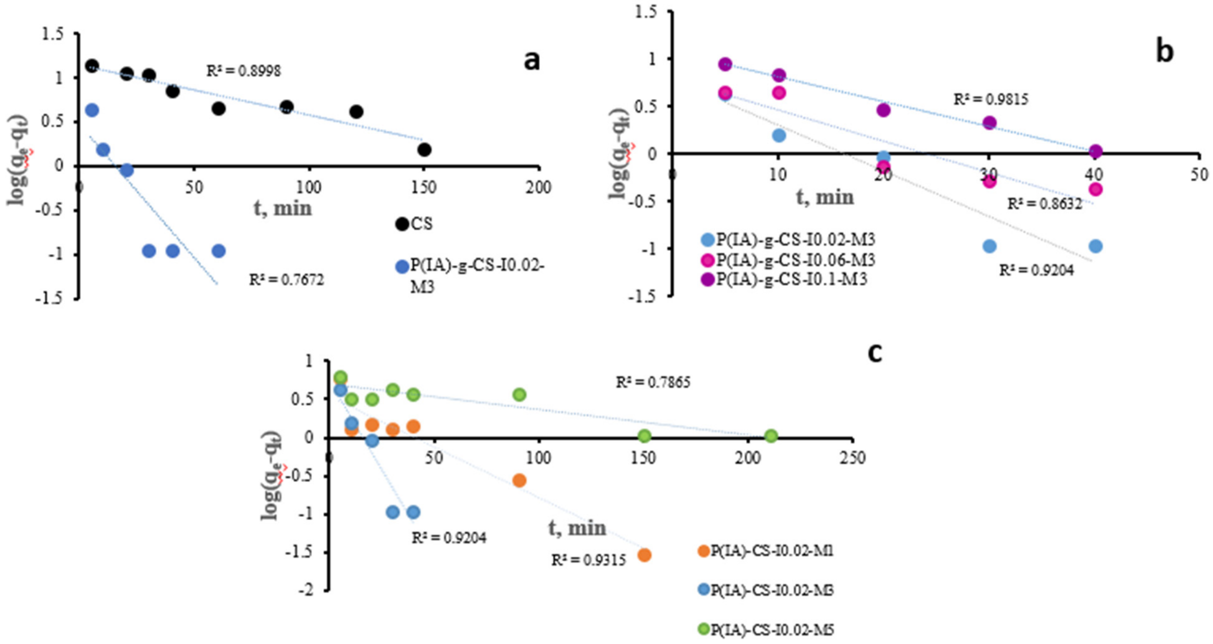

Figure S2 Pseudo-first order kinetic plots showing the effect of a) grafting, b) initiator concentration, and c) monomer concentration on the adsorption kinetics.

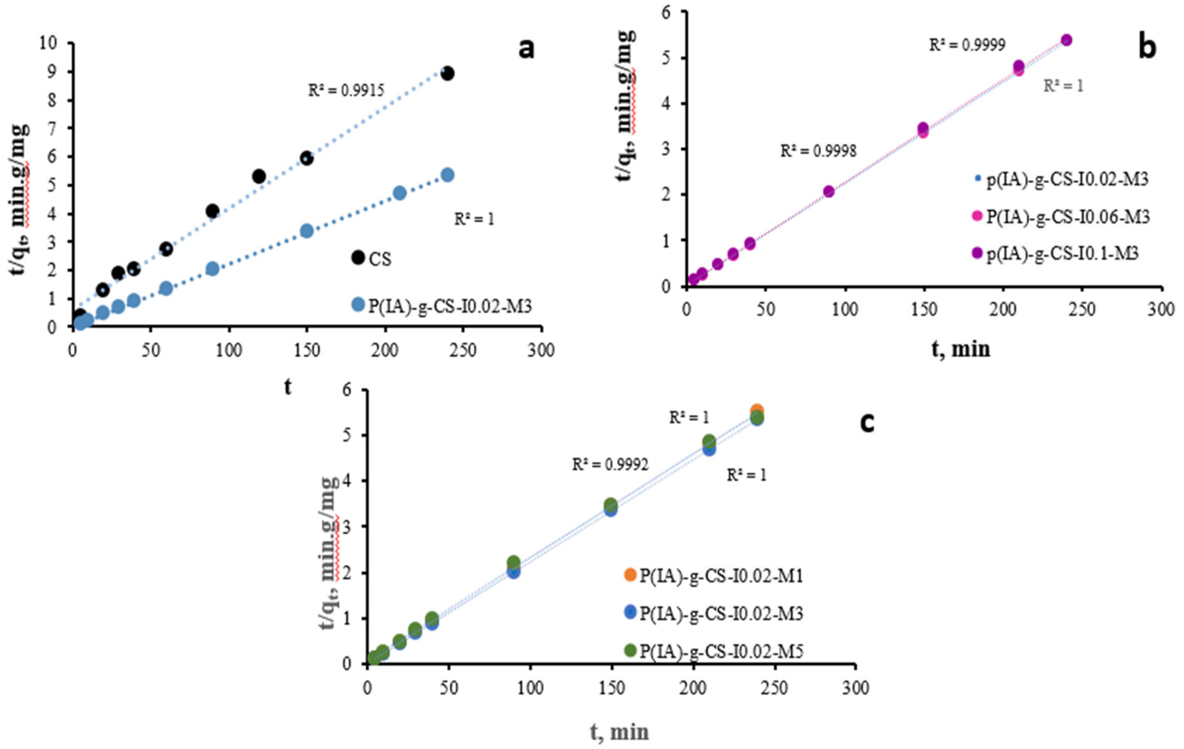

Figure S3 Pseudo-second order kinetic plots showing the effect of a) grafting, b) initiator concentration, and c) monomer concentration on the adsorption kinetics.

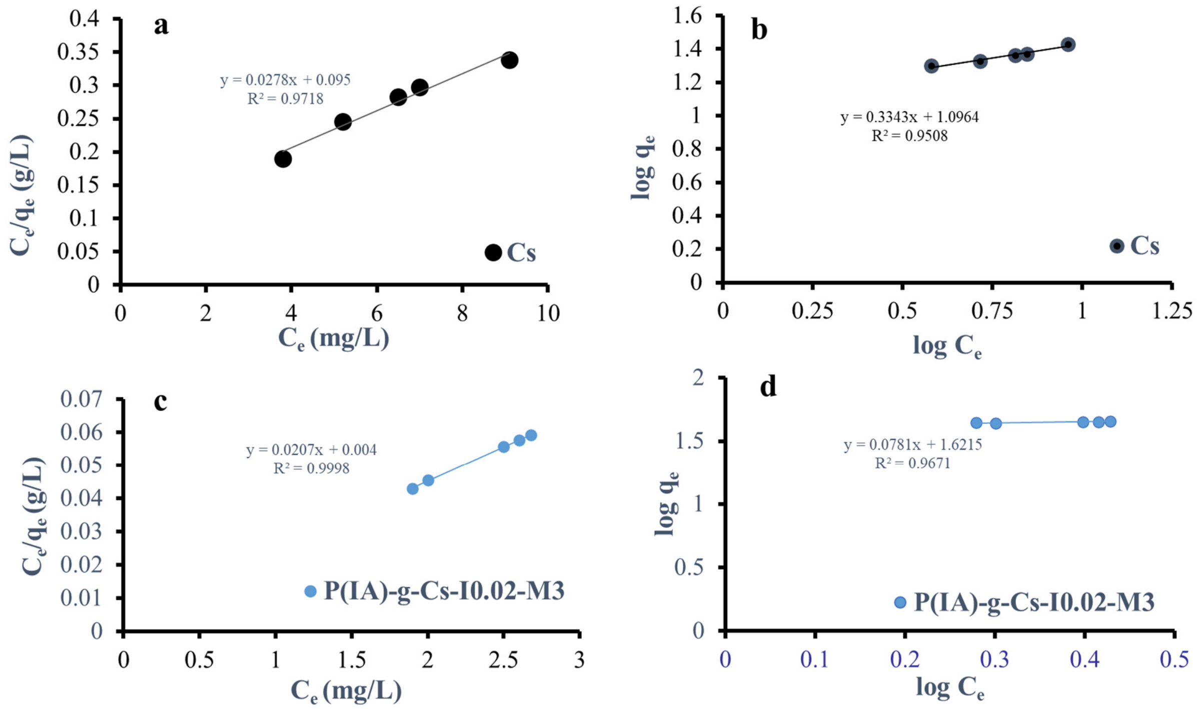

Figure S4 Langmuir and Freundlich respective plots for CS (a, b) and P(IA)-g-CS-I0.02-M3 (c, d).

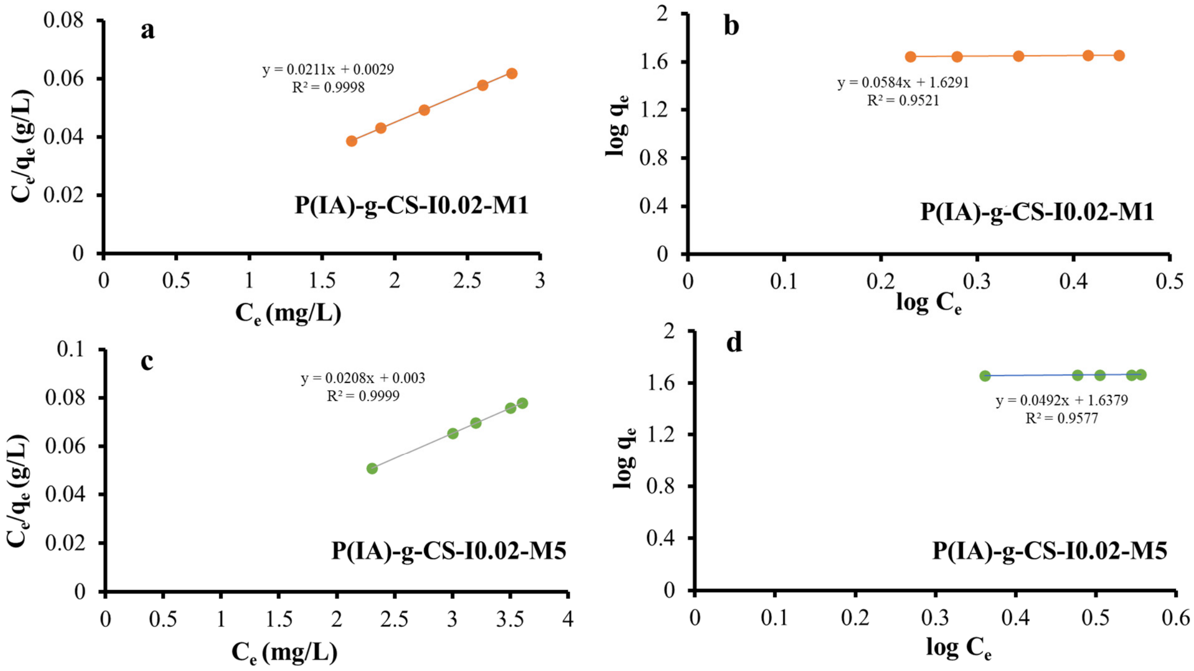

Figure S5 Langmuir and Freundlich respective plots for grafted polymers with monomer amounts of M1 (a, b) and M5 (c, d).

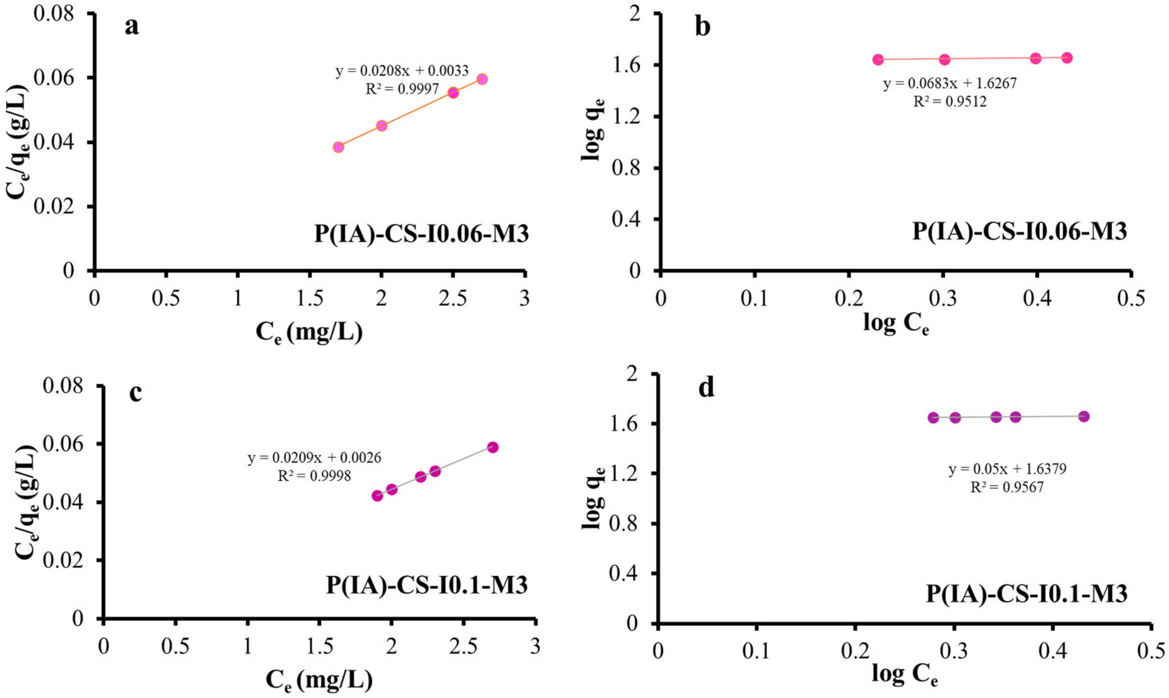

Figure S6 Langmuir and Freundlich respective plots for grafted polymers with initiator amounts of 0.06 (a, b) and 0.1 (c, d).

References

[1] Abdel Salam, J., Saleh, A.A.; El Nenaiey, T.T., Yang, H., Shoeib, T., El-Sayed, M.M.H. Mono- and multi-component biosorption of caffeine and salicylic acid onto processed cape gooseberry husk agri-food waste. *ACS Omega*, 2023, 8, 20697-20707.

[2] Farghal, H., Nebsen, M., El-Sayed, M.M.H. Multifunctional chitosan/xylan-coated magnetite nanoparticles for the simultaneous adsorption of the emerging contaminants Pb(II), salicylic acid, and congo red dye. *Water*, 2023, 15, 829.

[3] Farghal, H., Nebsen, M., El-Sayed, M.M.H., Eco-friendly biopolymer/activated charcoal magnetic nanocomposites with enhanced stability and adsorption properties for water treatment applications, Accepted, *J. Polym. Environ.*, 2023.

[4] Simonin, J.-P., On the comparison of pseudo-first order and pseudo-second order rate laws in the modeling of adsorption kinetics. *Chem. Eng. J.*, 2016. 300, 254-263.

[5] El-Sayed, M.M.H., Elsayed, R.E., Attia, A., Farghal, H. H., Azzam, R. A. and Madkour, T. M., Novel nanoporous membranes of bio-based cellulose acetate, poly(lactic acid) and biodegradable polyurethane *in-situ* impregnated with catalytic cobalt nanoparticles for the removal of methylene blue and congo red dyes from wastewater. *Carbohydr. Polym. Technol. Appl.*, 2021, 2, 100123.
